# Co-existing of adenoid cystic carcinoma and invasive squamous cell carcinoma of the uterine cervix: a report of 3 cases with immunohistochemical study and evaluation of human papillomavirus status

**DOI:** 10.1186/s13000-015-0376-z

**Published:** 2015-08-19

**Authors:** Xiaohua Shi, Shafei Wu, Zhen Huo, Qing Ling, Yufeng Luo, Zhiyong Liang

**Affiliations:** Department of Pathology, Peking Union Medical College Hospital, Chinese Academy of Medical Sciences and Peking Union Medical College, No. 1 Shuaifuyuan Road, Beijing, 100730 Dongcheng District P.R. China

## Abstract

**Backgrounds:**

The aim of this study was to describe the clinicopathological characteristics and high-risk human papillomavirus (HPV) infection status in patients diagnosed with co-existing of adenoid cystic carcinoma (ACC) and invasive squamous cell carcinoma (SCC) of the uterine cervix.

**Methods:**

Three patients were identified from the pathology databank of Peking Union Medical College Hospital from year 2000 to 2014. Immunohistochemistry and *in situ* hybridization (ISH) were employed in this study.

**Results:**

The patients were aged 64, 77 and 63 years (average, 68 years old). All the patients were postmenopausal women who presented with bloody or watery vaginal discharge. The cervical cytology screening results were all suspicious for high-grade squamous intraepithelial lesion (HSIL). The subsequent cervical colposcopy biopsies all showed cervical intraepithelial neoplasia III (CINIII). One patient received only a cervical conization, whereas the other two patients underwent hysterectomy. The immunohistochemical results showed that the ACC compartments were positive for CK7 and CD117; the cases of SCC were negative for CK7 and CD117. P63 staining was strongly positive and diffuse throughout the SCC compartments, whereas only patchy positive areas were observed in the ACC. MYB exhibited strong nuclear staining in the ACC and SCC compartments but negative staining in the endocervical gland. *In situ* hybridization (ISH) signals for high-risk HPV DNA and mRNA were present in the two compartments of all three patients. The patients had no evidence of disease at an average follow-up time of 21.6 months.

**Conclusion:**

High-risk HPV was present in both the ACC and SCC compartments in all three patients.

## Background

ACC of the uterine cervix was first described by McGee et al. in 1965 [1], and there have been more than 100 cases documented in the literature, most of which have been published as case reports. ACC is an uncommon variant of adenocarcinoma of the uterine cervix and accounts for only 1 % of all cases of primary cervical adenocarcinoma [2]. Compared to its counterpart in the salivary gland, ACC is believed to have a worse prognosis in the uterine cervix. ACC is thought to behave more aggressively than SCC, but because of its rarity, a standard treatment method has not been established; patients with ACC receive the same treatment as those with SCC [3]. ACC has been reported to be associated with intraepithelial neoplasia (CIN), invasive squamous cell carcinoma, adenocarcinoma and sarcoma in the uterine cervix [4], but the relationship between ACC and these tumors has not been investigated.

HPV plays an important role in the pathogenesis of cervical cancer, and the association between HPV and squamous cell carcinoma or adenocarcinoma has been well established. Most previous studies have focused on exploring the relationship between HPV and squamous intraepithelial neoplasia, squamous cell carcinoma, adenocarcinoma or small cell carcinoma [5, 6]; few have reported the association between HPV and ACC in the uterine cervix.

Here, we present 3 cases of co-existing of ACC and SCC in the uterine cervix and describe the immunohistochemical characteristics and high-risk HPV infection status based on an analysis of HPV DNA and mRNA expression by *in situ* hybridization. To the best of our knowledge, this is one of the first and largest studies of its kind to explore the immunophenotypic properties and HPV status of the cervix.

## Methods

### Patients’ information

The surgical pathology archives and consultation practices of Peking Union Medical College Hospital from year 2000 to 2014 were searched using the terms “adenoid cystic carcinoma” and “cervix”. Only 4 cases were identified, 3 of which were ACC combined with SCC, and case 1 has been reported previously [7]; the other case of the 4 was co-existing of ACC and ABC. Clinicopathological characteristics were collected, including age, symptoms, tumor size and type, tumor growth pattern, surgery, FIGO stage, therapy and prognosis. This study was approved by the Institutional Review Board of Peking Union Medical College Hospital.

### Immunohistochemistry

All the immunohistochemical studies were performed on formalin-fixed, paraffin-embedded tissue sections with a thickness of 4 μm using standard autostaining protocols on a Ventana Benchmark XT autostainer (Ventana Medical Systems Inc., Tucson, AZ). For AE1/AE3, Calponin, CK7 and SMA, the positive signals appeared as tan particles in the cytoplasm. For CD117, tan granules on the membrane or in the cytoplasm indicated positive staining. For P63, P53, TTF-1, MYB and Ki-67, tan particles in the nucleolus were observed in positive samples. Tan particles were not detected in the corresponding locations in negative samples.

### Human papillomavirus DNA detection by *in situ* hybridization

For the *in situ* detection of high-risk HPV infection at the DNA level, biotin-labeled HPV probe solutions (Leica, Newcastle, UK) were applied to formalin-fixed, paraffin-embedded tissue sections. The examination was carried out automatically using the BOND HPV Probe set that captures HPV subtypes 16, 18, 31, 33 and 51 using the Leica BOND-MAX system (Bond Ready-to-Use ISH HPV Probe, CAT # PB0829). Cases with brown punctate and/or diffuse signals in the tumor cell nuclear or cytoplasm were interpreted as positive.

### Human papillomavirus mRNA detection by RNA *in situ* hybridization

For the *in situ* detection of high-risk HPV integration at the mRNA level, the RNAscope FFPE 2.0 HD detection kit (Brown) (Advanced Cell Diagnostics, Hayward, CA, USA) was used according to the manufacturer’s instructions. Briefly, 2- to 3-mm thick FFPE tissue sections were deparaffinized, heated, treated with a protease and hybridized with the probe at 40 °C for 2 h (HPV High Risk 7 Pool: 16,18, 31, 33, 35, 52 and 58, CAT # 312351, Probe Name: Probe - HPV HR7). After washing and amplification, target RNA was detected with 3,39-diaminobenzidine. Nuclei were counterstained with hematoxylin. Positive staining was indicated by brown punctate dots in the cytoplasm.

## Results

The patients were 64, 77 and 63 years old (average, 68 years old). All the patients were postmenopausal women; the first two patients suffered from bloody vaginal discharge, and the other patient visited a doctor because of watery vaginal discharge. The results of the cervical ThinPrep cytological test were all suspicious of high-grade squamous intraepithelial lesion (HSIL). The cervical examination of two patients revealed exophilic and polypoid nodules with maximum diameters of 5 and 15 mm; the tumors were hemorrhagic and fragile, and they bled heavily in response to palpation. The other patient’s cervical mucosa was smooth with no evidence of a tumor. The subsequent cervical colposcopy biopsies all revealed cervical intraepithelial neoplasia III (CINIII) with no evidence of invasive squamous carcinoma; the biopsy specimens were too superficial to make a determination of invasive squamous carcinoma. One of patients only underwent a cervical conization and received a final diagnosis of ACC with concurrent invasive SCC. The surgical margin was positive. Pelvic magnetic resonance imaging and positron emission tomography-computed tomography (PET-CT) were performed after surgery, and these examinations revealed no evidence of residual disease or local metastasis. The patient refused to undergo any further operations and therefore received only radiation and chemotherapy. The other two patients underwent a hysterectomy, and the results showed that the tumors were confined to the uterus. The margins were negative. One patient received radiation and chemotherapy, whereas the other received only radiation therapy. The patients were all clinical stage I at the time of diagnosis, and there was no evidence of recurrence or metastasis at the follow-ups at 20 or 25 months. The demographic and clinical characteristics of the patients and their treatment regimens are presented in Table 1.

**Table 1:** Clinical characteristics of cases of coexisting ACC and SCC

Case	Age (years)	Tumor size (mm)	Tumor type	Cervical smear findings	Treatment	Margins	Clinical stage	Post-surgery treatment	Recurrence or metastasis	Follow up (months)/outcome
1	64	5	Polypoid	HSIL	Conization	+	I	R + C	-	20, NED
2	63	10	Invasive	HSIL	Hysterectomy	-	I	R + C	-	25, NED
3	77	15	Polypoid	HSIL	Hysterectomy	-	I	R	-	20, NED
					Salpingectomy					
					Oophorectomy					

*HSIL* high-grade squamous intraepithelial lesion, *NA* not available, *R* radiation, *C* chemotherapy, *NED* no evidence of disease

**Fig. 1:**
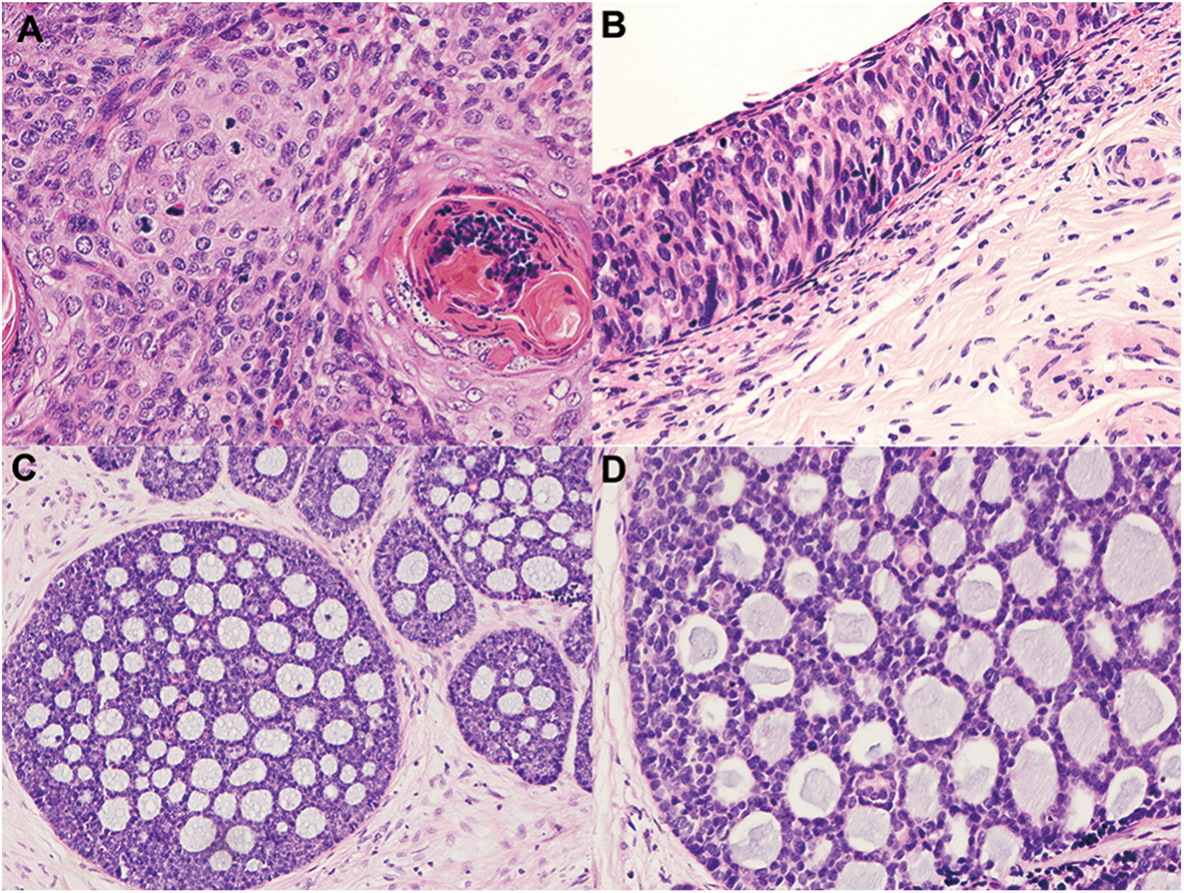
**a** The invasive SCC component of the tumor with keratinization (HE, ×40); **b** CINIII in the overlaying squamous epithelial (HE, ×40); **c** and **d** The lower power and high power image of ACC component of the tumor (HE, ×40; ×100)

The squamous cell carcinoma morphology was demarcated from the adenoid cystic carcinoma in the cervix (Fig. 1a). The invasion depth of the tumor was 10, 11 and 9 mm. The third one showed lymphovascular invasion. The proportions of the SCC compartments in the three cases were 60, 80 and 80 % (Table 2). Cervical SCC is thought to originate from intraepithelial neoplasia, and all the cases presented with CINIII (Fig. 1b). Two of the three patients had poorly differentiated SCC, and the other patient had well differentiated SCC (Table 2). Unlike SCC which exhibits keratinization, nuclear enlargement, cell atypia and a fibroblastic reaction around the tumor cells, the ACC compartment was characterized by nests of cells surrounded by basement membrane components. The basaloid tumor cells in the ACC formed a cribriform growth pattern composed of both ductal and abluminal myoepithelial cells (Fig. 1c). The basaloid tumor cells had a small to moderate amount of cytoplasm, and the nuclei were uniform without conspicuous nucleoli (Fig. 1d).

**Table 2:** Histopathological and immunohistochemimal findings of cases of coexisting ACC and SCC

Case/Component		Histopathology				Immunohistochemistry							ISH	
		Differentiation or growth pattern	Proportion (%)	Depth of invasion (mm)	LVI	CD117	CK7	SMA	Calponin	P63	MYB	P16	DNA	mRNA
1	SCC	Morderate	60	9	-	-	-	-	-	D+	+	+	+	+
	ACC	Cribriform	40			+	+	+	+	P+	+	+	+	+
2	SCC	Low	80	11	-	-	-	-	-	D+	+	+	+	+
	ACC	Cribriform	20			+	+	+	-	P+	+	+	+	+
3	SCC	Low	80	10	+	-	-	-	-	D+	+	+	+	+
	ACC	Cribriform	20			+	+	+	-	P+	+	+	+	+

*ISH in situ* hybrization, *LVI* lymphovascular invasion, *D* diffuse, *P* patchy

**Fig. 2:**
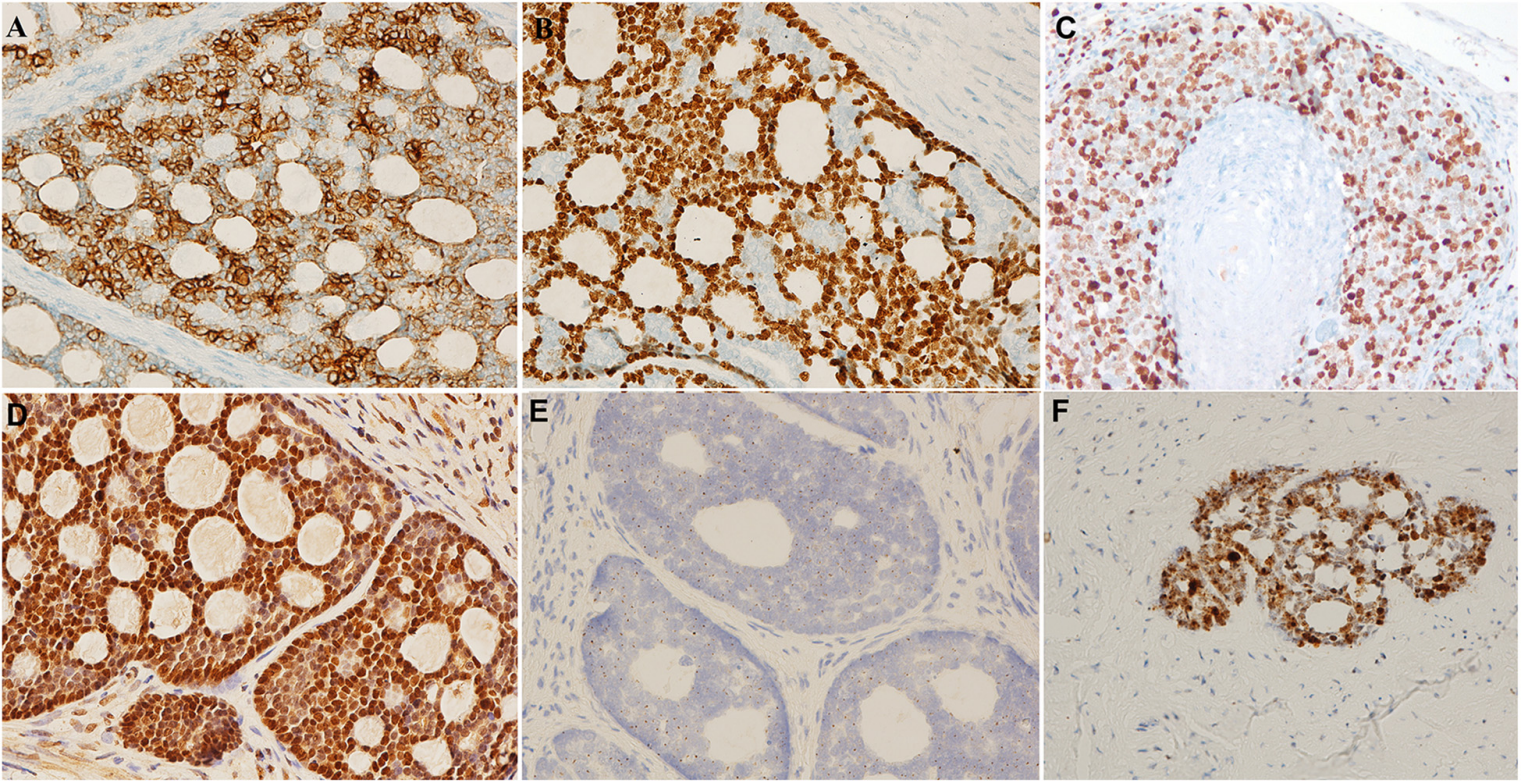
**a** CK7 immunostain is positive in the ACC component (CK7, ×40); **b** P63 immunostain is patchy positive in the ACC component (P63, ×40); **c** P63 immunostain is diffuse positive in the SCC component (P63, ×40); **d** C-MYB immunostain is positive in the ACC component (C-MYB, ×40); **e** HPV detected by DNA ISH method is punctate positive in the ACC component (HPV ISH, ×100); **f** HPV detected by mRNA ISH method is positive in the ACC component (HPV ISH, ×100)

The pathological characteristics, immunohistochemical staining and high-risk HPV detection results are summarized in Table 2. The two tumor compartments in the 3 cases were positive for AE1/AE3, and the ACC compartment was positive for CK7 (Fig. 2a) and CD117; the SCC compartment was negative for CK7 and CD117. The SCC compartment in all the cases exhibited strong and diffuse positive staining for P63 (Fig. 2b), whereas only patchy positive staining was observed in the ACC compartment; these patches generally appeared in the peripheral cells (Fig. 2c). In all the cases, SMA was expressed in the myoepithelial cells in the ACC compartment, but not the SCC compartment. MYB nuclear staining was strong in the ACC and SCC compartments (Fig. 2d) but was negative in the endocervical gland. P16, which is thought to be a surrogate marker for the presence of HPV, exhibited strong, diffuse nuclear and cytoplasmic reactivity in both the ACC and SCC compartments in all 3 cases. HPV DNA *in situ* hybridization signals were present in the two compartments of all 3 cases: 1 case showed punctate nuclear staining, whereas the other two cases exhibited punctate and diffuse nuclear staining (Fig. 2e). The same results were obtained from the HPV mRNA ISH analysis (Fig. 2f): strong brown cytoplasmic staining was present in the two compartments in all 3 cases. The hybridization signals were distributed throughout the basaloid and ductal cells in the ACC as well as in both the surface dysplasia and the invasive component of the SCC.

## Discussion

In this study, we presented three cases of co-existing of ACC and SCC in the uterine cervix. In the literature review which was presented in Table 3, there were few reports of co-existing of ACC and SCC and scarce data on the HPV status in these cases. The average age of the three patients in this study was 68 years, which was consistent with the patients described in the literature (average, 61.4 years old). The majority of the patients were postmenopausal women, and the predominant presenting symptom was vaginal bleeding, which was the case in two of our patients. The tumors usually present as a polypoid or outgrowth mass in the cervix; more advanced clinical stage (stage II and III) is associated with early recurrence and metastasis. Including our three cases, high-risk HPV infection has been identified in 6 of the 8 cases in both the ACC and SCC compartments, whereas HPV infection was absent in two of the eight cases in both compartments.

**Table 3:** Summary of reported cases of cervical adenoid cystic carcinoma coexisting with invasive squamous cell carcinoma

Author	Age	Symptom	Tumor type/size	Clinical stage	HPV result	Microscopic appearance	Treatment method	Status(duration of survival)
Grayson, W et al. [16]	68	NA	NA	NA	HPV16 in both	ACC + SCC + CIS + sarcoma	NA	NA
	55	NA	NA	NA	HPV16 in both	ACC + SCC + CIS + focal ABC + sarcoma	NA	NA
	36	NA	NA	NA	Neg	ACC + SCC + sarcoma	NA	NA
Yang, Y. J et al. [17]	36	NA	No gross nodular	NA	Neg	ACC + SCC + CIS + condyloma	Surgery	No
Grayson, W et al. [18]	NA	NA	NA	NA	HPV16 in both	ACC + superficially invasive SCC	NA	NA
Seth, A et al. [19]	24	Bleeding	Exophytic/3 cm	NA	NA	ACC + focal SCC	Surgery	NA
Grayson, W et al. [4]	NA	NA	NA	NA	NA	6 of 18 ACC showed ACC + invasive SCC, 1 case showed ACC + invasive SCC + ABC	NA	NA
Mathoulin-Portier, M. P et al. [20]	73	Bleeding	Necrotic mass/5 cm	NA	NA	ACC+ a RARE focal SCC + sarcoma	Surgery	Metastasis 12 months later, Live with disease 36 months
Manhoff, D. T et al. [21]	80	Bleeding	Protruded mass/NA	NA	NA	ACC + focally invasive SCC+ malignant stroma	Surgery + Radiation	NED, 6 months
Albores-Saavedra et al. [22]	NA	NA	NA	NA	NA	3 of 7 patients showed ACC + focal invasive SCC	NA	NA
Ferry, J. A et al. [23]	NA	NA	NA	NA	NA	1 of 14 patients showed ACC + SCC + CIS + ABC	NA	NA
Musa, A. G et al. [24]	NA	NA	NA	NA	NA	2 of 17 patients showed ACC + invasive SCC	NA	NA
Berchuck, A et al. [25]	72	Bleeding	Exophytic/9.5 cm	FIGO IIIB	NA	ACC+ large cell SCC	Surgery + Radiation	NED, 6 months
Mazur, M. T et al. [26]	76	Spotting	No visible tumor/NA	NA	NA	ACC + small foci of invasive SCC + CIS	Surgery + Radiation	NA
Miles, P. A et al. [27]	NA	NA	NA	NA	NA	3 of 12 showed ACC+ invasive SCC	NA	NA
Hoskins, W. J et al. [28]	54	NA	NA	FIGO IB	NA	Adenosquamous carcinoma and the adeno component is ACC	Surgery + Radiation	NED, 12 months
Bittencourt, A. L et al. [29]	60	Bleeding	Fragile mass/7 cm	FIGO IB	NA	ACC + invasive SCC	Surgery + Radiation	NED, 9 months
	72	Bleeding	NA	FIGO IB	NA	ACC + invasive SCC	Radiation	NED, 27 months
Tchertkoff and Sedlis [30]	63	Bleeding	NA	NA	NA	ACC + invasive SCC	Surgery	Metastasis 3 years later live with disease
Fowler, W. C et al. [31]	NA	NA	NA	NA	NA	1 of 9 patient showed ACC+ invasive SCC	NA	NA
Shingleton, H. M et al. [32]	78	Bleeding	Fungated mass/NA	FIGO III	NA	ACC+ focal SCC	Radiation	DOD, 3 months
	65	Spotting	NA	FIGO IIB	NA	ACC+ focal SCC	Surgery + Radiation	NED
	64	NA	NA	NA	NA	ACC+ poorly differentiated SCC	Surgery + Radiation	DOD, 5 months
Ramzy, I et al. [33]	68	Bleeding	No visible tumor	NA	NA	ACC + invasive SCC	Surgery + Radiation	DOD, 27 months

*NA* not available, *ACC* adenoid cystic carcinoma, *SCC* squamous cell carcinoma, *CIS* carcinoma *in situ*, *ABC* adenoid basal carcinoma, *NED* no evidence of disease, *DOD* died of disease

Immunohistochemistry was helpful for distinguishing SCC from ACC in these cases. P63 is a useful marker that shows strong and diffuse nuclear staining in SCC but only peripheral or compartmentalized distribution in ACC. SCC is negative for CK7 and CD117, but the luminal component of ACC is positive for CK7 and CD117. ACC is characterized by the expression of myoepithelial markers, such as SMA, S-100 or calponin and there was positive SMA staining in the ACC from the three cases in our study. Recent studies have shown that a t(6;9)(q22–23;p23–24) translocation that results in a MYB-NFIB gene fusion is specific for ACC [8, 9]; this MYB-NFIB fusion contributes to MYB overexpression. In this study, the ACC in all three cases exhibited strong nuclear MYB staining, which provided further confirmation of the diagnosis.

The major differentiation of ACC is ABC, which is also a neoplasia that originates from the salivary gland. The discrimination of ACC from ABC basically relies on morphology; ACC exhibits cellular pleomorphism, mitoses, necrosis and stromal hyalinization. Besides these characteristics, ABC is defined based on the absence of the MYB translocation and CD117 immunostaining. All of our 3 cases showed CD117 positive staining by IHC, while in Andrzej Wincewicz’s study, ABC was negative for CD117 [10]. ACC is thought to be more aggressive than SCC in the cervix and to have a high potential for metastasis and recurrence, whereas ABC is considered to be a neoplasia instead of a carcinoma because of its indolent character and benign clinical behavior. Lin reported a case of co-existing of ABC and SCC in the cervix and concluded that we must avoid overestimating the clinical stage because ABC is thought to be a benign lesion [11]. However, ACC is thought to behave more aggressively than SCC; therefore, the proper identification of ACC is important, and an appropriately enhanced treatment regimen, including post-operative radiation and chemotherapy, along with careful clinical follow-up are recommended.

The association between HPV and cervical squamous cell carcinoma and adenocarcinoma has been well documented, but few articles have investigated the relationship between HPV and ACC of the cervix. Currently, over 100 genotypes of HPV have been identified, and they can be subdivided into three groups: high-risk, low-risk and unclassified [12]. The high-risk HPV types include types 16, 18, 31, 33, 35, 45, 51, 52 and 56, and these types are believed to play a major role in the pathogenesis of cervical carcinoma. The progression from a premalignant lesion to cervical cancer is primarily caused by high-risk HPV, especially types 16 and 18 [13]. Once HPV integrates into the host genome, the E2 opening read frame (ORF) is deleted, resulting in the deregulated expression of the viral oncogenes E6 and E7; these two oncogenes interfere with critical cell cycle pathways (p53 and RB) and increase cell proliferation [12, 14]. P16 is considered to be a surrogate detection marker for HPV infection in the cervix. P16 is negatively regulated by Rb, and it is therefore over-expressed in cervical carcinoma cells in which Rb is inactivated by HPV E7. There are several methods for detecting HPV status, for example, non-isotopic *in situ* hybridization (NISH) and PCR; NISH can identify both episomal and integrated HPV in archival cervical biopsy material with a sensitivity of 2.5-20 viral copies per cell. The NISH signal pattern was analyzed using the criteria established by Cooper et al. There are three types of signals: type 1 (diffuse), indicative of episomal HPV DNA; type 2 (punctate); and type 3 (a combination of punctate and diffuse), indicative of integrated HPV DNA. In our study, one of the three cases showed a type 2 signal, and the other two showed type 2 and 3 signals, which was indicative of HPV integration into the host genome in both the SCC and ACC compartments. mRNA ISH is emerging as a promising method for detecting HPV infection based on the mRNA levels in archival materials; this method not only analyzes the signal *in situ* but also determines the status of E6/E7, which are thought to be vital for the pathogenesis of cervical cancer, with high sensitivity. In our three cases, both the SCC and ACC compartments were positive based on the NISH results.

The origination of ACC in the cervix remains uncertain, but some immunohistochemical evidence has shown that this lesion may originate from the reserve cell layer of the cervical epithelium [4]. The reserve cells lie beneath the columnar mucinous epithelial cells of the endocervix and are considered to have the ability to generate both the squamous and glandular epithelium. To the best of our knowledge, the histogenesis of concurrent ACC and SCC has not yet been studied, and whether they undergo polyclonal origination to form a merged tumor or they are monoclonal in origin, sharing a common stem cell, needs to be explored further. HPV is reported to be an early event that is detected not only in invasive carcinoma but also in intraepithelial neoplasia [15]. In our study, it was demonstrated that the integration of high-risk HPV and the up-regulation of the viral oncogenes E6/E7 occurred in the ACC, SCC and CIN. These data may suggest that these diseases are of monoclonal origin and that the divergence occurs late in the tumorigenic process.

## Conclusion

We present three cases of co-existing of ACC and SCC in the uterine cervix. High-risk HPV was detected in both the ACC and SCC in all three cases. Because ACC is an aggressive form of cervical cancer with a propensity for local recurrence and widespread metastasis, the identification of ACC and the enhancement of post-operative treatment regimens, including radiation and chemotherapy, are critically important and careful follow-up is recommended. However, the number of the cases in our study was small, and the clinical follow-up was limited; more cases are necessary to further explore the histogenesis of ACC in the uterine cervix.

## References

[1] McGee JA, Flowers CE, Tatum BS (1965). Adenoid cystic carcinoma of the cervix: report of a case. Obstet Gynecol.

[2] Vuong PN, Neveux Y, Schoonaert MF, Guettier C, Houissa-Vuong S (1996). Adenoid cystic (cylindromatous) carcinoma associated with squamous cell carcinoma of the cervix uteri: cytologic presentation of a case with histologic and ultrastructural correlations. Acta Cytol.

[3] Kaur P, Khurana A, Chauhan AK, Singh G, Kataria SP, Singh S (2013). Adenoid cystic carcinoma of cervix: treatment strategy. J Clin Diagnostic Res.

[4] Grayson W, Taylor LF, Cooper K (1999). Adenoid cystic and adenoid basal carcinoma of the uterine cervix: comparative morphologic, mucin, and immunohistochemical profile of two rare neoplasms of putative ‘reserve cell’ origin. Am J Surg Pathol.

[5] Mazarico E, Gomez-Roig MD, Minano J, Cortes L, Gonzalez-Bosquet E (2014). Relationship of human papilloma virus multiple genotype infection with patient's age and type of cervical lesion. Eur J Gynaecol Oncol.

[6] Horn LC, Lindner K, Szepankiewicz G, Edelmann J, Hentschel B, Tannapfel A (2006). p16, p14, p53, and cyclin D1 expression and HPV analysis in small cell carcinomas of the uterine cervix. Int J Gynecol Pathol.

[7] Shi X, Chang X, Wu H, Ren X, Liu T, Bui MM (2014). Co-existing adenoid cystic carcinoma and invasive squamous cell carcinoma of the uterine cervix: a rare case report and literature review. Ann Clin Lab Sci.

[8] West RB, Kong C, Clarke N, Gilks T, Lipsick JS, Cao H (2011). MYB expression and translocation in adenoid cystic carcinomas and other salivary gland tumors with clinicopathologic correlation. Am J Surg Pathol.

[9] Brill LB, Kanner WA, Fehr A, Andren Y, Moskaluk CA, Loning T (2011). Analysis of MYB expression and MYB-NFIB gene fusions in adenoid cystic carcinoma and other salivary neoplasms. Mod Pathol.

[10] Wincewicz A, Lewitowicz P, Urbaniak-Wasik S, Kanczuga-Koda L, Koda M, Adamczyk-Gruszka O (2014). Adenoid basal carcinoma-like tumor combined with invasive squamous cell carcinoma foci of uterine cervix - a case report of 55-year-old woman with literature review. Rom J Morphol Embryol.

[11] Lin YC, Perng CL, Chang YM, Li YF, Tsai YM, Wu GJ (2013). Coexistent squamous cell carcinoma and adenoid basal carcinoma in the uterine cervix and infection with human papillomavirus (HPV 31). Taiwan J Obstet Gynecol.

[12] Munoz N, Bosch FX, de Sanjose S, Herrero R, Castellsague X, Shah KV (2003). Epidemiologic classification of human papillomavirus types associated with cervical cancer. N Engl J Med.

[13] Lax S (2011). Histopathology of cervical precursor lesions and cancer. Acta Dermatovenerol Alp Panonica Adriat.

[14] Kalantari M, Karlsen F, Kristensen G, Holm R, Hagmar B, Johansson B (1998). Disruption of the E1 and E2 reading frames of HPV 16 in cervical carcinoma is associated with poor prognosis. Int J Gynecol Pathol.

[15] Ueda Y, Enomoto T, Miyatake T, Ozaki K, Yoshizaki T, Kanao H (2003). Monoclonal expansion with integration of high-risk type human papillomaviruses is an initial step for cervical carcinogenesis: association of clonal status and human papillomavirus infection with clinical outcome in cervical intraepithelial neoplasia. Lab Invest.

[16] Grayson W, Taylor LF, Cooper K. Carcinosarcoma of the uterine cervix: a report of eight cases with immunohistochemical analysis and evaluation of human papillomavirus status. Am J Surg Pathol. 2001;25:338-47. 10.1097/00000478-200103000-00008 11224604

[17] Yang YJ, Gordon GB. Cervical adenoid cystic carcinoma coexisting with multiple human papillomavirus-associated genital lesions. A common etiology? Gynecol Obstet Invest. 1999;47:272-7. 10.1159/000010121 10352392

[18] Grayson W, Taylor L, Cooper K. Detection of integrated high risk human papillomavirus in adenoid cystic carcinoma of the uterine cervix. J Clin Pathol. 1996;49:805-9. 10.1136/jcp.49.10.805 PMC500773 8943745

[19] Seth A, Agarwal A. Adenoid cystic carcinoma of uterine cervix in a young patient. Indian J Pathol Microbiol. 2009;52:543-5. 10.4103/0377-4929.56158 19805968

[20] Mathoulin-Portier MP, Penault-Llorca F, Labit-Bouvier C, Charafe E, Martin F, Hassoun J, et al. Malignant mullerian mixed tumor of the uterine cervix with adenoid cystic component. Int J Gynecol Pathol. 1998;17:91-2. 10.1097/00004347-199801000-00018 9475200

[21] Manhoff DT, Schiffman R, Haupt HM. Adenoid cystic carcinoma of the uterine cervix with malignant stroma. An unusual variant of carcinosarcoma? Am J Surg Pathol. 1995;19:229-33. 10.1097/00000478-199502000-00012 7832282

[22] Albores-Saavedra J, Manivel C, Mora A, Vuitch F, Milchgrub S, Gould E. The solid variant of adenoid cystic carcinoma of the cervix. Int J Gynecol Pathol. 1992;11:2-10. 10.1097/00004347-199201000-00002 1373414

[23] Ferry JA, Scully RE. "Adenoid cystic" carcinoma and adenoid basal carcinoma of the uterine cervix. A study of 28 cases. Am J Surg Pathol. 1988;12:134-44. 10.1097/00000478-198802000-00007 2449087

[24] Musa AG, Hughes RR, Coleman SA. Adenoid cystic carcinoma of the cervix: a report of 17 cases. Gynecol Oncol. 1985;22:167-73. 10.1016/0090-8258(85)90023-x 2996993

[25] Berchuck A, Mullin TJ. Cervical adenoid cystic carcinoma associated with ascites. Gynecol Oncol. 1985;22:201-11. 10.1016/0090-8258(85)90028-9 2996994

[26] Mazur MT, Battifora HA. Adenoid cystic carcinoma of the uterine cervix: ultrastructure, immunofluorescence, and criteria for diagnosis. Am J Clin Pathol. 1982;77:494-500. 10.1093/ajcp/77.4.494 6176118

[27] Miles PA, Norris HJ. Adenoid cystic carcinoma of the cervix. An analysis of 12 cases. Obstet Gynecol. 1971;38:103-10. 4327155

[28] Hoskins WJ, Averette HE, Ng AB, Yon JL. Adenoid cystic carcinoma of the cervix uteri: report of six cases and review of the literature. Gynecol Oncol. 1979;7:371-84. 10.1016/0090-8258(79)90115-x 221309

[29] Bittencourt AL, Guimaraes JP, Barbosa HS, Carvalho WA, Lange RK, Barata AS, et al. Adenoid cystic carcinoma of the uterine cervix. Report of six cases and review of the literature. Acta Med Port. 1979;1:697-706. 233192

[30] Tchertkoff V, Sedlis A. Cylindroma of the cervix. American journal of obstetrics and gynecology. 1962;84:749-52. 10.1016/0002-9378(62)90025-x 13920034

[31] Fowler WC, Jr., Miles PA, Surwit EA, Edelman DA, Walton LA, Photopulos GJ. Adenoid cystic carcinoma of the Cervix. Report of 9 cases and a reappraisal. Obstet Gynecol. 1978;52:337-42. 212702

[32] Shingleton HM, Lawrence WD, Gore H. Cervical carcinoma with adenoid cystic pattern: a light and electron microscopic study. Cancer. 1977;40:1112-21. 10.1002/1097-0142(197709)40:3<1112::aid-cncr2820400321>3.0.co;2-m 198087

[33] Ramzy I, Yuzpe AA, Hendelman J. Adenoid cystic carcinoma of uterine cervix. Obstet Gynecol. 1975;45:679-83. 10.1097/00006250-197506000-00018 1143730

